# Nano-Pulse Stimulation Treatment Inhibits Pan02 Murine Pancreatic Tumor Growth and Induces a Long-Term Adaptive Immune Response with Abscopal Effects When Combined with Immune-Enhancing Agents

**DOI:** 10.1089/bioe.2024.0011

**Published:** 2024-06-12

**Authors:** Amanda McDaniel, Kristin Von Rothstein, Dacia Gonzalez, Richard Nuccitelli

**Affiliations:** Biology Department, Pulse Biosciences, Inc., Hayward, California, USA.

**Keywords:** nano-pulse stimulation, pancreatic cancer, regulated cell death, immunotherapy, co-stimulation, CD8

## Abstract

Pancreatic cancer is associated with a poor prognosis and immunotherapy alone has not demonstrated sufficient efficacy in the treatment of nonresectable tumors. Nano-Pulse Stimulation™ therapy (NPS™) applies nanosecond electric pulses that lead to regulated cell death, exposing tumor antigen to the immune system. To establish a primary Pan02 tumor, mice were intradermally injected with Pan02 cells into the right flank. Secondary, rechallenge tumors and distal, secondary tumors (abscopal response) were established by injecting Pan02 cells into the opposite left flank. After 5 days of tumor growth, one of the tumors was treated with NPS, followed by injection with an immune-enhancing agent to stimulate an immune response. Growth of the treated primary tumor and untreated rechallenge tumors (injected 60-days post-treatment) or distal secondary tumors (injected simultaneously with the primary) was monitored. NPS in combination with the adjuvant and TLR agonist, resiquimod (RES), was the optimal treatment regimen for both eliminating a primary Pan02 tumor as well as inhibiting growth of a Pan02 cell rechallenge tumor. This inhibition of the rechallenge tumor injected 2 months after eliminating the primary tumor suggests a long-term immune response had been stimulated. Additional support for this came from the observations that depleting CD8+ T-cells reduced inhibition of rechallenge tumor growth by 35% and rechallenge tumors had 3-fold more CD8+ T-cells than tumors injected after surgical resection of the primary tumor. When the NPS-treated tumor was immediately injected with the anti-OX40 antibody to agonize the function of the costimulatory T cell receptor, OX40, up to 80% of untreated abscopal tumors were eliminated. NPS plus RES was the most effective at both eliminating a primary tumor and inhibiting a rechallenge tumor. NPS treatment followed by injection of aOX40 was the most effective at inhibiting the growth of an untreated abscopal tumor.

## Introduction

Pancreatic ductal adenocarcinoma (PDAC) is associated with a poor prognosis that has led it to be classified as one of the deadliest cancers across the globe, and it is predicted to become the second leading cause of cancer-related death by the year 2030.^1^ An estimated 62,210 new cases will be diagnosed in the coming year, leading to an estimated 49,830 deaths. The 5-year survival rate for those diagnosed with pancreatic cancer between the years 2012 and 2018 is only 11.5%. In addition, over 52% of pancreatic tumors already show dissemination to distal sites within the peritoneal cavity upon diagnosis and, even when tumors are localized to the pancreas, approximately 30–40% of these are unresectable due to the local advancement of pancreatic tumor cells into surrounding tissues and blood vessels.^2,3^ These features of PDAC have led to a challenging path for researchers and physicians alike as few therapies show significant efficacy, including those that have demonstrated high efficacy in the treatment of other advanced cancers.^4,5^

Nano-Pulse Stimulation™ (NPS) therapy is a bioelectric modality that uses ultrashort electric pulses in the nanosecond range to initiate regulated cell death (RCD), exposing antigen to the immune system. NPS induces a transient permeabilization of the plasma and organelle membranes of targeted cells causing stress that alters the functioning of both the endoplasmic reticulum (ER) and mitochondria. Dependent upon the level of cellular damage, NPS will either initiate a cascade of events that leads to RCD or commit the cell to death directly through necrosis.^6^ Although necrosis immediately exposes the immune system to cellular contents, RCD serves to provide further signals to the immune system to surveil and inspect the contents of dying cells for the presence of abnormal antigens in order to launch the most appropriate immune response.^6–9^ In addition to tumor cell death, examination of the site of the tumor post-treatment using flow cytometry has shown that NPS will destroy any cell type residing in a tumor, including tumor-resident immune cells such as tumor-associated macrophages (TAMs), myeloid-derived suppressor cells (MDSCs), and T regulatory cells (Tregs), thus disrupting the tumor microenvironment (TME) and depleting it of immunosuppressive cell types.^10^ NPS has demonstrated significant efficacy at breaking apart the immunosuppressive environment of a tumor, clearing a primary tumor and exposing tumor antigen to the immune system as observed in a variety of murine syngeneic models.^10–16^ NPS demonstrated 100% efficacy in eliminating 4T1 murine breast cancer,^16^ 75% elimination rate after NPS treatment of mouse hepatocellular carcinomas,^17^ and a 80–90% response rate in the treatment of rat hepatocellular carcinomas.^18^ Response rates are related to tumor type and size, as well as treatment energy. This is noteworthy as it demonstrates that the therapeutic properties of NPS extend beyond providing cell death signals and exposing antigens; NPS also causes the actual depletion of the physical and immunological barriers within pancreatic tumors. These properties are what made us believe that NPS may be uniquely qualified to treat the dense immunosuppressive environment of a pancreatic tumor.

Pan02 mouse tumor cell line was utilized as a translational model of human pancreatic tumors^19,20^ due to its generation of a similarly immunoresistant TME and lack of pre-existing T cell immunity due to inadequate antigen presentation. Pan02 tumor cells were derived from C57BL/6 mice, so it is syngeneic with this strain and the injected tumors are not rejected. Molecular and cellular screening of Pan02 tumors have shown that they contain a very small number of infiltrating T cells and that those that do reside are either exhausted and/or lack effector functions, such as granzyme B secretion and interferon (IFN)-γ production, rendered them unable to launch an effective cytotoxic attack on tumor cells.^21^ Pan02 tumors, like human PDAC tumors, contain several immunosuppressive cell subsets, including Tregs,^22–24^ MDSCs,^24,25^ and TAMs,^26^ and express a variety of inhibitory proteins and signaling molecules, including transforming growth factor-β,^27^ programmed cell death ligand 1 (PD-L1),^24^ and cytotoxic T-lymphocyte–associated antigen 4. In addition, they have a similarly high resistance to most chemotherapeutic agents, and their potential for metastasis is significant.^28^ These features make Pan02 an appropriate model to investigate the ability of NPS to disrupt the pancreatic tumor environment and deplete it of immunosuppressive cells while also exposing tumor antigen to the outside immune system, to effectively traffic and prime antitumor CD8+ T cells.

In this work we examined the ability of NPS to 1) clear a primary tumor; 2) inhibit the growth of a rechallenge tumor when used alone as a single agent and in combination with resiquimod (RES), a Toll-like receptor (TLR) 7/8 agonist; and 3) to inhibit the growth of untreated abscopal tumors when used in combination with aOX40. TLR agonists have a strong binding affinity to receptor sites meant to recognize foreign pathogens and this actively boosts immune signaling more than endogenous cell death signals.^29,30^ After tumor antigen is released and taken up by dendritic cells (DCs), RES further promotes adaptive immune recognition by binding to TLR7 and TLR8 on DCs, stimulating the production of type 1 IFNs and helping to prime and expand the population of tumor-specific CD8+ T cells.^31,32^

While rechallenge experiments are key for demonstrating that an adaptive response has been generated, they provide little direct translational information about how the treatment would benefit human therapy. Because 52% of pancreatic tumors are found to have metastasized by the initial diagnosis, we also wanted to examine the ability of NPS to induce a real-time abscopal immune response that could cause regression of distal tumor sites. We therefore used a two-tumor model in which one tumor was left untreated so that we could observe possible abscopal effects. We found that the addition of aOX40 to NPS treatment had a synergistic effect that is capable of both permanently clearing a primary Pan02 tumor and causing the complete regression of most secondary distal tumors.

## Materials and Methods

### In vivo tumor model

#### Mice

Female C57BL/6J mice, 6–8 weeks old (Jackson Laboratories, Sacramento, CA), were acclimated for at least 3 days before treatment, housed in groups of 10, and both flanks were shaved before the start of tumor inoculations. Animals were maintained on a 12-h light/ dark cycle. Water (Milli-Q) and food (Pirolab Diet 20 chow) were given *ad libitum*. All experiments and research staff training for animal handling and care were performed in accordance with animal care guidelines set forth by the Pulse Biosciences IACUC. All tumor cell injections and treatments were conducted under isoflurane inhalation anesthesia. If tumors grew to 1000 mm^3^, the animal was euthanized immediately. For euthanasia, the animal was given an overdose of isoflurane anesthesia followed by cervical dislocation. Most treated tumors were ablated, and those animals lived for up to 90 days before euthanizing. Tumor size and animal health were monitored twice per week and less than 1% of the mice died before meeting the criteria for euthanasia. All animal experiments were approved by the Pulse Biosciences IACUC. All treatments were performed under isoflurane inhalation anesthesia.

#### Tumor cells

The Pan02 tumor line was obtained from the NIH and propagated in tissue culture with Dulbecco’s modified Eagle medium supplemented with 10% v/v fetal bovine serum (FBS), penicillin/streptomycin, and harvested for inoculation between passages 9 and 12 for all studies.

#### Rechallenge experiments

**FIG. 1.**
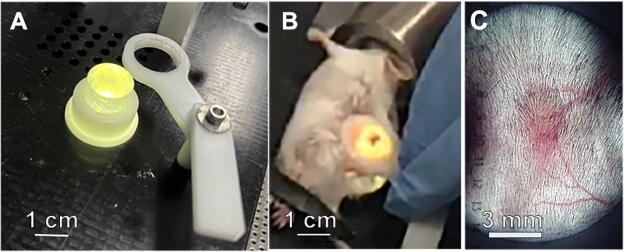
Method for treating tumors with NPS. **(A)** Platform with silicone light post for clamping skin that is enclosing the tumor to be treated. **(B)** Mouse under isoflurane inhalation anesthesia with skin tumor pulled over the silicon light post to visualize the tumor. **(C)** Image of a Pan02 tumor on the light post that is treated by surrounding it with two parallel rows of needles that penetrate the skin and apply bipolar pulses.

**FIG. 2.**
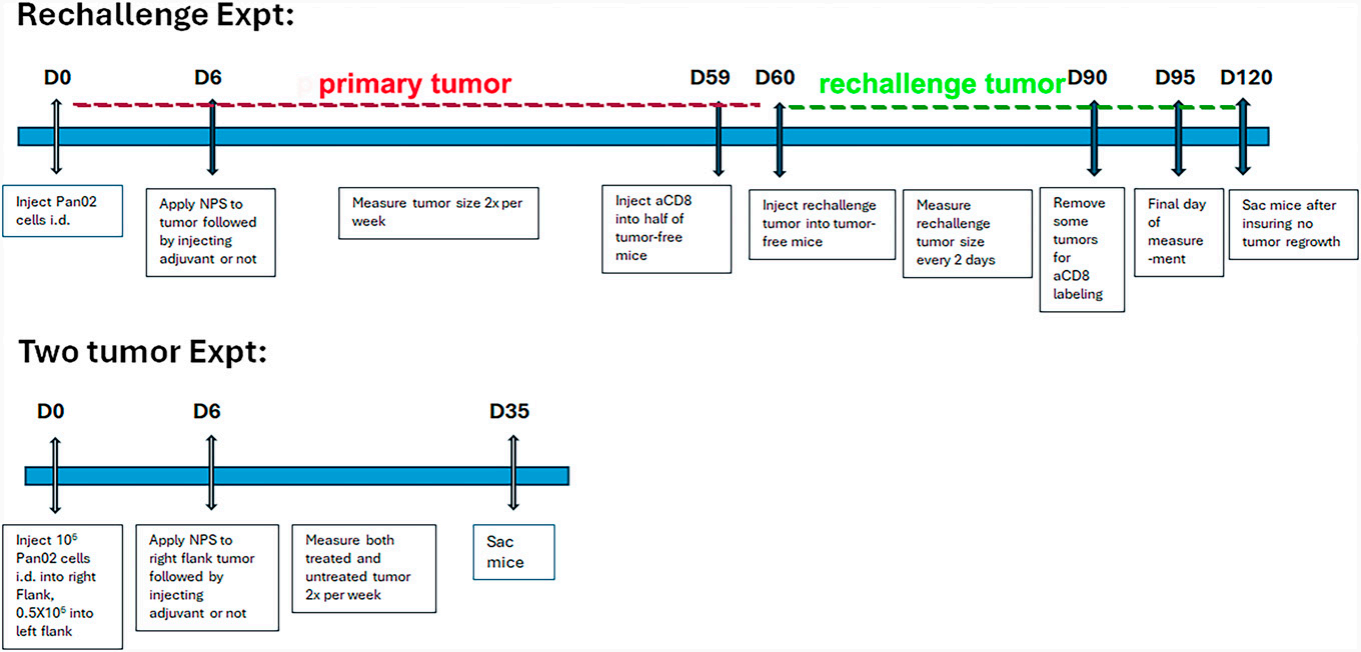
Timeline of the two experiments described in the Methods section.

As shown in Fig. 1, primary tumors were initiated in mice by intradermal (i.d.) injection into the right flank with 1 × 10^6^ Pan02 cells/30 μL in Hank’s balanced salt solution (HBSS) while the mice were under isoflurane anesthesia. Tumors were injected into the intradermal space within the skin so that they could be stretched over a platform designed to isolate them from the body and internal organs for treatment (Fig. 1). Tumor growth was measured twice a week with calipers. The volumes were determined using the formula, volume = length × width^2^/2. Tumors were randomized to treatment groups when the largest diameter reached ∼5mm on Day 6 post tumor inoculation. Mice whose tumors were not eliminated or had returned were sacrificed on Day 60. One control that we included was to surgically remove (resect) the primary tumor to observe any immune response due to the presence of the tumor alone. We included a tumor injection and measurement timeline for clarity (Fig. 2).

Mice that remained tumor-free 60 days after inoculation remained in the study for rechallenge. To determine if the tumor inhibition occurring after NPS (mid-dose) + RES was mediated by the infiltration of CD8+ cytotoxic T cells, half of the mice who received this treatment to eliminate their primary tumors received an intraperitoneal dose of an anti-CD8 antibody to antagonize the function of CD8+ T cells 1 day before they were rechallenged with Pan02 tumor cells (D59).

Mice that remained in the study were inoculated with another 1 × 10^6^ cells/30 μL (i.d. in HBSS) (Rechallenge Day 0) along with a group of mice that were completely naive to tumor antigen. Inoculation sites were monitored for tumor growth twice per week. Tumor volumes were measured and recorded until Rechallenge Day 35 (Study Day 95) for analysis. Mice who developed tumors remained in the study until the final day of measurement (Study Day 95). Mice whose tumor regrowth was fully prevented remained in the study until Study Day 120 to ensure no tumor regrowth.

#### Abscopal experiments

On study day zero (D0), primary tumors to be targeted directly with a treatment were initiated in mice by injection of Pan02 cells (1 × 10^6^/30 μL HBSS, i.d.) into the right flank. Distal secondary tumors to be observed for an abscopal effect were initiated by injection of Pan02 cells (0.5 × 10^6^/30 μL HBSS, i.d.) into the contralateral left flank. We used half as many cells for the secondary tumors because we wanted them to grow more slowly than the primary treated tumors. Both primary and distal secondary tumors were monitored for regression and elimination until Day 35. Growth of both primary and secondary tumors was measured and recorded twice per week.

#### Tumor treatments

Mice were anesthetized by breathing 1.2% isoflurane in oxygen and individually placed onto the treatment platform. Six days post-inoculation, mice that developed tumors were treated with NPS energy delivered by the CellFX™ System (Pulse Biosciences, Hayward, CA). NPS therapy was approved for the treatment of benign skin lesions by the FDA in 2021 and has been used to remove skin lesions on more than 5000 patients with excellent results and no adverse reactions. Upon completion of the procedure, mice were returned to their home cage for recovery.

NPS therapy was delivered using a 5.0 × 5.0 × 3.5 mm treatment tip attached to a handpiece plugged into the CellFX^®^ pulse generator. The treatment tip contained two parallel rows of five microneedles 3.5 mm in length, and the rows were spaced 5 mm apart. Mouse tumors were treated by stretching the skin containing the tumor over a lighted translucent silicone treatment post and inserting the probe needles through the skin to enclose the tumor (Fig. 1). We treated the tumors 6 days after tumor cell injection because that was when the tumors had grown to 5 mm in diameter and were the ideal size for treatment with the 5 × 5 mm treatment tip. We tested two injection times for the injection of immune stimulants into the tumors and found that injecting right after NPS treatment produced far better immune system stimulation than waiting 24 hours to inject.

#### Immune-enhancing agents

We piloted a range of known immune system modifiers, including the Toll-like receptor agonists, CpG, RES, and imiquimod,^33^ and members of the tumor necrosis factor (TNF) superfamily crucial to T cell costimulation such as OX40.^34,35^ OX40 is induced on the T cell surface some hours after recognition of antigen, and antibodies to OX40 provide signals to a T cell to allow prolonged cell division after activation and to prevent excessive cell death. Immune-enhancing agents were each injected intratumorally (i.t.) at a volume of 20 μL using a 0.5 mL syringe/28G, into primary tumors either immediately after NPS treatment or 1 day later.

RES (TLR7/8): Lyophilized RES (InvivoGen R484; cat# tlrl-484) was reconstituted with 1 mL physiological grade water to a working concentration of 5 mg/mL (50 μg/10 μL). An additional 10 μL of PBS was added for a total injection volume of 20 μL/tumor (i.t.) for RES-only injections.

CpG (TLR9): Lyophilized CpG oligonucleotide (ODN1826; Eurofins Genomics) was reconstituted with 1 mL of phosphate-buffered saline (PBS) to a working concentration of 5 mg/mL (50 μg/10 μL). An additional 10 μL of PBS was added for a total injection volume of 20 μL/tumor (i.t.) for CpG only injections.

Anti-OX40 (OX40 agonist): Anti-OX40 antibody (Bio X cell; cat# BE0031) was diluted into PBS to a working concentration of 5 mg/mL (50 μg/10 μL) from stock solution. An additional 10 μL of PBS was added for a total injection volume of 20 μL/tumor (i.t.) for aOX40-only injections.

Adjuvant (RES or CpG) ± aOX40 combinations: 10 μL of working concentration (50 μg/10 μL) adjuvant (RES or CpG) was combined with 10 μL working concentration aOX40 (50 μg/10 μL) for a 20 μL coinjection (1:1) of either RES + aOX40 or CpG + aOX40 (i.t.).

#### Anti-CD8 immunohistochemistry of tumor samples

Thirty days after injecting the rechallenge tumors, some of the rechallenged mice were sacrificed and a rectangle of skin (2.5 cm long by 1.5 cm wide) containing the intradermal Pan02 tumor in the center was excised, attached flat to a paper card stock without stretching, and submersed in 10% neutral-buffered formalin. After 24–48 h of fixation, each sample was bisected with the tumor directly in the center, samples were marked with surgical ink to maintain orientation, and both halves were embedded in paraffin. Samples were processed for paraffin histology (AcePix, Hayward, CA) and sectioned to a thickness of 5 μm. Samples were stained with anti-mouse CD8 antibody for recognition of CD8+ T cells in tumor samples.

#### ImageJ thresholding of aCD8-stained slide images for particle measurement

All scanned aCD8-stained immunohistochemistry (IHC) slide images were zoomed to 2X in a slide imaging program, and each 2X image was saved with a 1000 μm scale bar on the lower left side. Images were then opened in the ImageJ program, and the scale bar was used to set the pixel to μm ratio. A ratio of 123:1000 pixels to μm was determined, and the scale was set. The image was then isolated from the background by tracing the boundaries of the image using the freehand tool for measurement and analysis. After the tumor image boundaries were set, the image was then isolated from the background to measure only the tumor area, measured using the scaling parameters and then converted to grayscale. The threshold was set so that the number of particles was approximately equivalent to the number of CD8+ particles in the original image, and then the number of CD8+ particles was measured in the ImageJ program and exported to excel. The CD8+ particles/cm^2^ ratio was calculated in Excel by dividing the number of particles within the tumor image by the area of the tumor. The CD8+ particles/cm^2^ for the images were compared between groups using a Kruskal–Wallis one-way analysis of variance (ANOVA).

### Statistical analyses of tumor efficacy data

Statistical analyses were performed using GraphPad Prism software (v9, La Jolla, CA). Tumor inhibition and elimination rates were compared between groups using a chi-square contingency test. One-way ANOVA was used to compare tumor volumes. A two-tailed *p* value of < 0.05 was considered statistically significant (*p < 0.05; **p < 0.01; ***p < 0.001; ****p < 0.0001).

## Results

### Primary tumor elimination and inhibition of rechallenge tumors using mid-dose NPS + RES

**FIG. 3.**
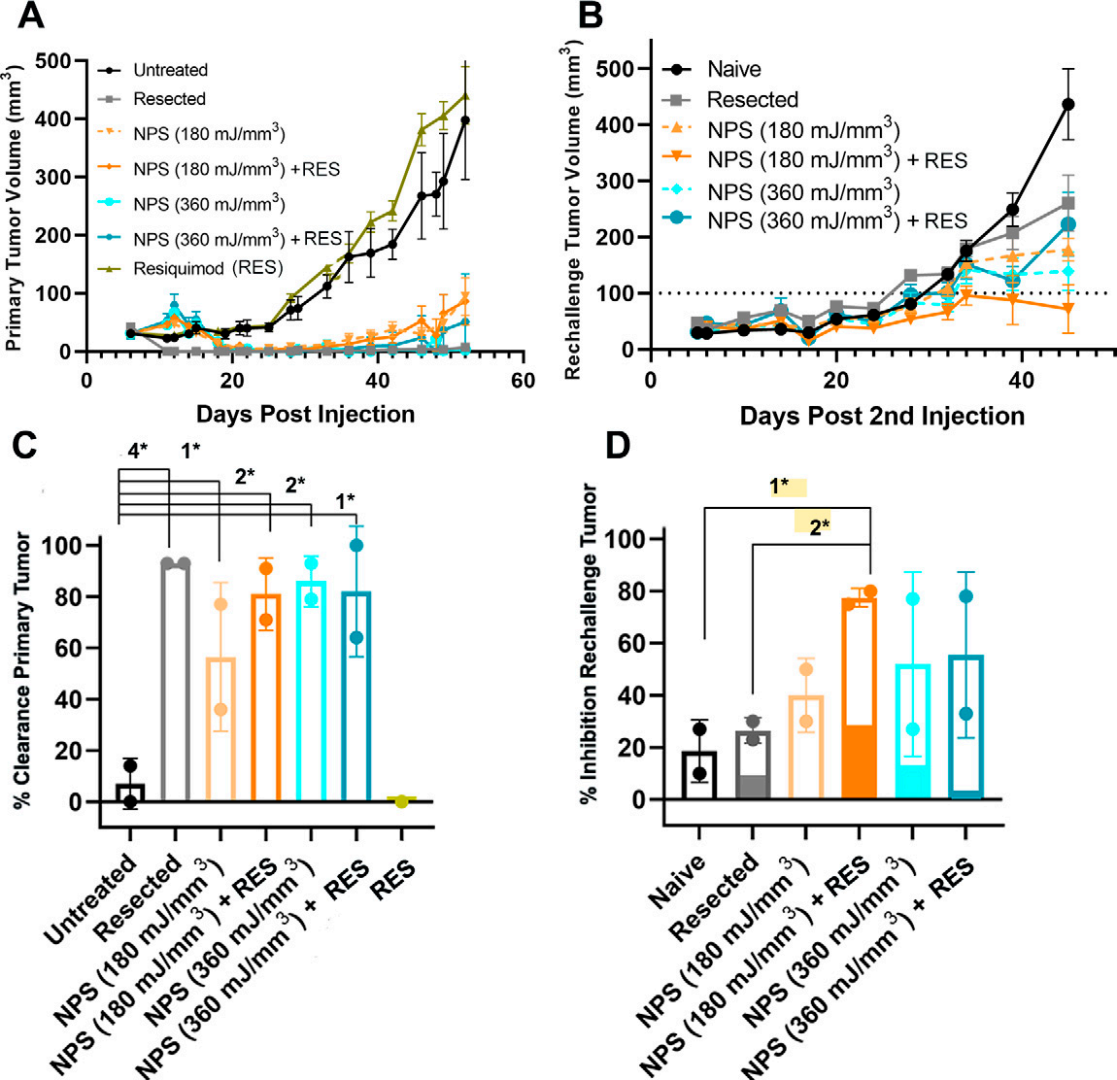
Primary and rechallenge tumor growth. Each data point represents the mean size of tumors in 10 mice. **(A)** Measurements of primary tumor volume following indicated treatments of NPS at 6 days post-inoculation with RES injection immediately after NPS. **(B)** Measurements of rechallenge tumors. Mice that remained tumor-free following initial clearance after treatment were rechallenged with a bolus of Pan02 cells 60 days after the initial primary tumor injection. Error bars in A and B represent SEM. **(C)** Percentage of primary tumors completely cleared by Day 60 postinjection in two separate experiments, each represented by a dot. NPS (180 mJ/mm^3^) alone only eliminated 57% of all primary tumors; however, when RES (50 µg; i.t.) was injected in combination with this mid-level dose of NPS, the combination eliminated 80% of all primary tumors. Injecting RES alone did not eliminate any tumors. **(D)** Percentage of rechallenge tumors that were inhibited (<100 mm^3^) by Day 32 post-rechallenge tumor injection in two separate experiments, each represented by a dot. The darkened portion of each bar represents the percentage of tumors that were completely inhibited, with no Pan02 tumor growth post-rechallenge injection. **(A)–(B)**. One-way ANOVA; **(C)–(D)** Chi-square contingency test. Significance denoted by asterisks: 1*p < 0.05; 2*p < 0.01; 3*p < 0.001; 4*p < 0.0001).

As shown in Fig. 3, preliminary studies indicated that mid-range NPS followed by the i.t. injection of RES was the most effective at inhibiting primary tumor growth, whereas injecting RES alone had no effect on tumor growth (Fig. 3A). Therefore, this control was not repeated in later experiments. Those mice that were tumor free at 60 days postinjection received a second (rechallenge) injection, and tumor growth was monitored (Fig. 3B). Rechallenge tumor growth was fastest in mice whose primary tumor was resected and slowest in mice whose primary tumor was treated with moderate energy levels of NPS plus RES (Fig. 3B). The partial inhibition of rechallenge tumor growth in mice with resected tumors is due to the small immune response resulting from the 5 days that the tumor was present before resection. The percentage of clearance of primary tumors by each treatment along with the percentage of inhibition of the rechallenge tumor is also presented (Fig. 3C, D). While mid-dose NPS (180 mJ/mm^3^) eliminated only 57% of all primary intradermal murine Pan02 pancreatic tumors when used alone, the rate of elimination increased to 81% when it was combined with a post-treatment injection of RES (50 μg; i.t.). This rate of primary tumor elimination was similar to that of a high dose of NPS (360 mJ/mm^3^) (86%) and high-dose NPS (360 mJ/mm^3^) + RES (82%) (Fig. 3C). However, only the mid-dose NPS + RES condition was capable of significantly inhibiting the growth of a rechallenge tumor relative to naive (*p* = 0.023) and resected (*p* = 0.0052) controls (Fig. 3D). The superior ability of the mid-dose NPS (180 mJ/mm^3^) + RES condition to inhibit or prevent growth of a rechallenge injection of Pan02 cells was indicative of a long-term adaptive memory response and prompted us to select this condition for additional study going forward.

**FIG. 4.**
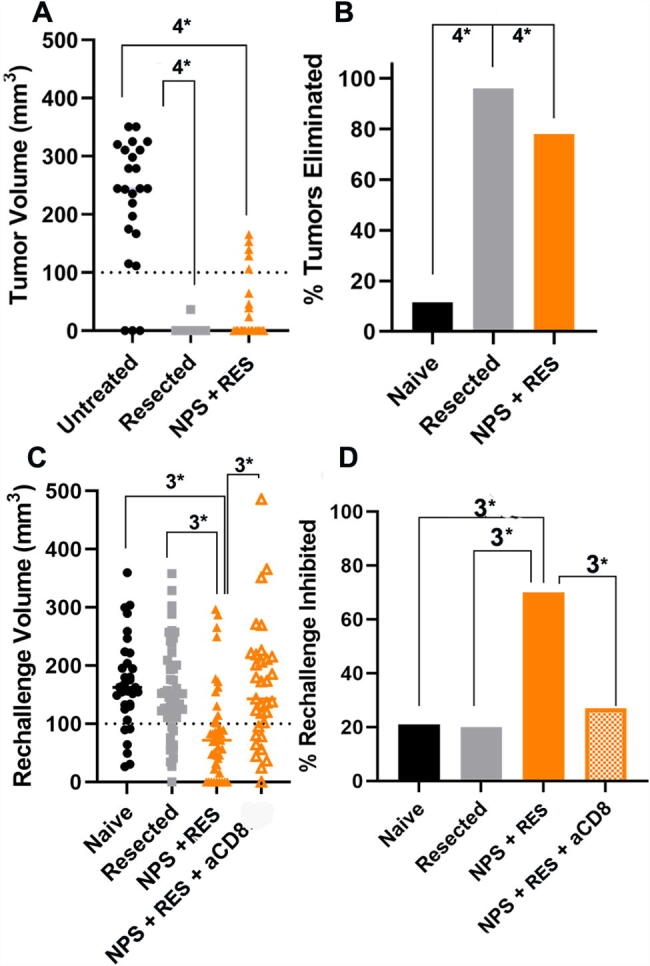
Distribution of treated tumor volumes at Day 52 post-inoculation and rechallenge tumor volumes on Day 34 post-rechallenge. **(A)** Statistical comparison of primary tumor volumes between Untreated, Resected, and NPS (180 mJ/mm^3^) + RES groups on Day 52. Tumor volumes were very significantly reduced (*p* < 0.0001) in both the Resected and NPS + RES groups relative to the Untreated condition but did not differ significantly from each other. **(B)** Percentage of primary tumors eliminated across all experiments by Day 52. The NPS (180 mJ/mm^3^) + RES condition eliminated approximately 80% of all primary tumors without reoccurrence (*p* < 0.0001). Although the rate of elimination after resection was higher (∼95%) the two did not differ significantly. (A = one-way ANOVA; B = Chi-square contingency tests [3*:*p* < 0.001; 4*:*p* < 0.0001]). **(C)** Rechallenge tumor volumes on Day 34 post-rechallenge. NPS (180 mJ/mm^3^) + RES rechallenge tumor volumes were significantly reduced relative to all control conditions: [Naive *p* = 0.001; Resected *p* = 0.003; NPS (180 mJ/mm^3^) + RES + aCD8 *p* = 0.003]. **(D)** Percentage of rechallenge tumors inhibited (<100 mm^3^) by Day 34. NPS (180 mJ/mm^3^) + RES inhibited 70% of all rechallenge tumors. This was significantly greater than Naive (21%; *p* < 0.0001); Resected (20%; *p* < 0.0001); and NPS (180 mJ/mm^3^) + RES + aCD8 (27%; *p* = 0.002). (Analyses: C = one-way ANOVA; D = Chi-square contingency test [3*: *p* < 0.001, 4*: *p* < 0.0001]).

As shown in Fig. 4, across all rechallenge studies, primary tumor volumes were significantly reduced after treatment with NPS (180 mJ/mm^3^) + RES relative to no treatment by D52 (Fig. 4A). NPS (180 mJ/mm^3^) + RES eliminated 78% of all primary tumors without recurrence (*p* < 0.0001) (Fig. 4B). Rechallenge tumor volumes were significantly reduced in the NPS (180 mJ/mm^3^) + RES group relative to all control conditions by D34 post-rechallenge inoculation: [naive *p* = 0.001; resected *p* = 0.003; NPS (180 mJ/mm^3^) + RES + aCD8 *p* = 0.003] (Fig. 4C). Mice whose primary tumor was eliminated by NPS (180 mJ/mm^3^) + RES demonstrated a 70% rate of rechallenge tumor inhibition (<100 mm^3^) across all studies. (Fig. 4B).

**FIG. 5.**
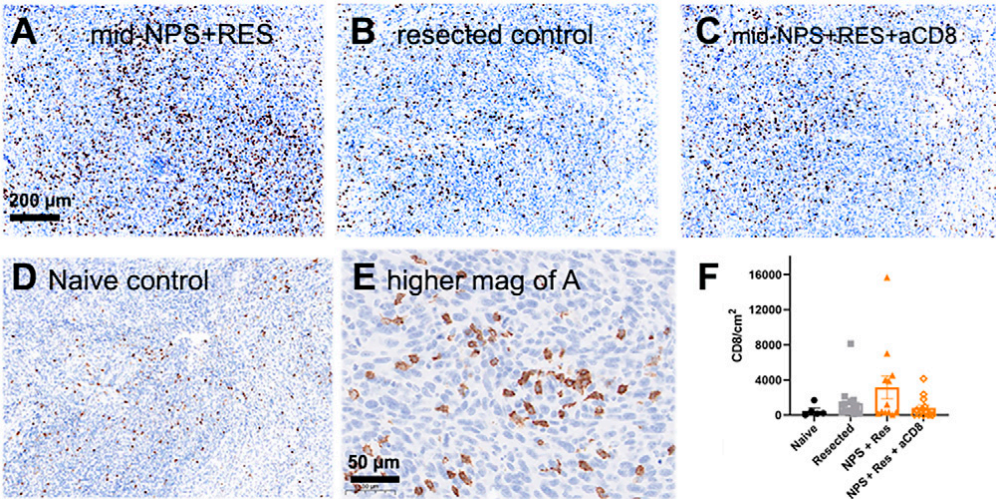
Immunohistochemistry of rechallenge tumors labeled with anti-CD8 antibodies (brown) 30 days postinjection. **(A)** Rechallenge tumor taken from mouse whose primary tumor was treated with mid-range NPS + RES injection immediately afterward (15,690 CD8/mm^2^). **(B)** Rechallenge tumor taken from mouse whose primary tumor was resected (2140 CD8/mm^2^). **(C)** Rechallenge tumor taken from mouse whose primary tumor was treated with mid-range NPS + RES with anti-CD8 antibodies injected IP into the mouse (4140 CD8/mm^2^). **(D)** Tumor taken from mouse who did not receive a prior injection of Pan02 cells (Naive) (180 CD8/mm^2^). **(E)** Higher magnification of a section from 3A. **(F)** Plot of the distribution of CD8 densities in four conditions indicated. Error bars represent SEM.

As shown in Fig. 5, IHC staining confirmed the increased presence of CD8-positive infiltrate in cells in rechallenge tumors. An anti-mouse CD8-antibody was used to stain rechallenge tumors to identify infiltrating CD8+ T cells (Fig. 5). When the primary tumors were treated with NPS (180 mJ/mm^3^) + RES, rechallenge tumors had more infiltrate that was CD8 positive (15,690 CD8/cm^2^), on average, than those depleted of CD8+ T cells (4140 CD8/cm^2^). Rechallenge tumors in mice from the NPS (180 mJ/mm^3^) + RES group had more invading CD8-positive infiltrate, than those in both the naive (507 CD8/cm^2^) and resected (1361 CD8/cm^2^) control groups (Fig. 5). However, while the average was higher and a trend observed, the high level of variability in the number of CD8+ cells within the NPS (180 mJ/mm^3^) + RES group prevented statistical significance.

### Two-tumor model to identify abscopal effects of NPS treatment with and without immune enhancement

**FIG. 6.**
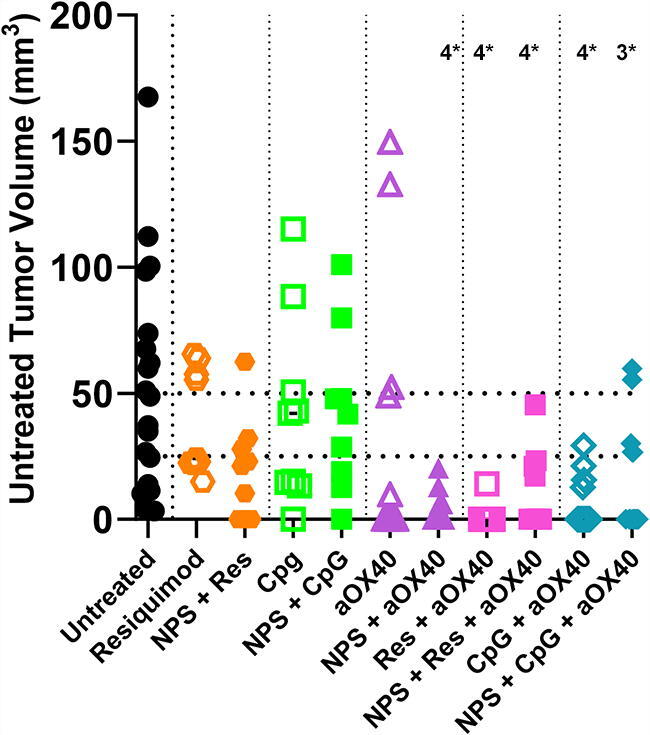
Distribution of untreated (UT) (abscopal) tumor sizes measured on day 35 post-inoculation. Injection into the primary tumor of 20 μg each of the immune stimulants, CpG, RES, or aOX40, alone had no significant effect on the untreated tumor size. However, the combination of 20 μg aOX40 injection i.t. following NPS (180 mJ/mm^3^) treatment of the primary tumor inhibited the untreated tumor growth more strongly than either treatment alone. The probabilities that these observed growth inhibitions of the untreated tumor were not significantly different from that measured in animals in which the primary tumor was not treated are indicated by the number of asterisks (4*: *p* < 0.0001; 3*: *p* < 0.001).

As shown in Fig. 6, we initially investigated the i.t. injection of the immune enhancers, RES, CpG, or aOX40 both alone and following NPS treatment and observed the strongest effect by aOX40. The use of either adjuvant (RES or CpG) alone or in combination with NPS (180 mJ/mm^3^) reduced the size of the untreated secondary tumor but did not reach a level of significance (Fig. 6). In contrast, when the costimulant aOX40 was added to any of these treatments, the size of the secondary tumor was reduced in size significantly: (Untreated (UT) vs NPS (180 mJ/mm^3^) + aOX40 *p* < 0.0005; UT vs Res + aOX40 *p* < 0.0001; UT vs NPS (180 mJ/mm^3^) + Res + aOX40 *p* < 0.0001; UT vs CpG + aOX40 *p* < 0.0001; UT vs NPS (180 mJ/mm^3^) + CpG + aOX40 *p* < 0.0006).

**FIG. 7.**
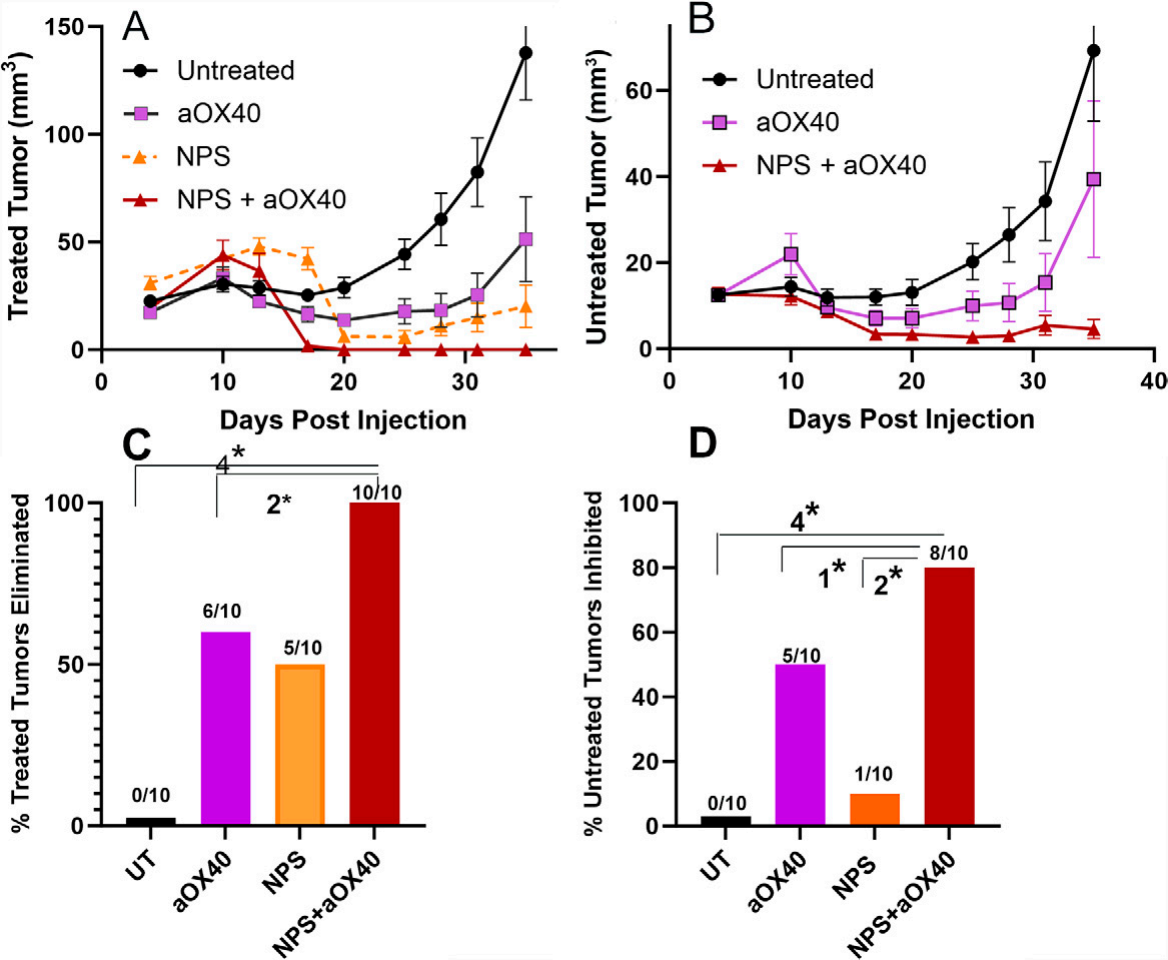
Treated and untreated tumor growth after treating the primary tumor with either NPS (180 mJ/mm^3^), aOX40 i.t. injection, or the combination of both. **(A)** Time course of treated tumor growth when the primary tumor was either untreated or treated with NPS (180 mJ/mm^3^), aOX40, or the combination of both. When aOX40 was combined with NPS the treated tumor growth was more strongly inhibited than with either alone. **(B)** Time course of untreated (abscopal) tumor growth following injection of the primary tumor with either 50 μg aOX40 or the combination of NPS treatment followed by 50 µg aOX40 i.t. injection. The combination treatment exhibited the strongest inhibition of untreated tumor growth. Error bars in A and B represent SEM. **(C)** Percentage of treated tumors eliminated by day 30. **(D)** Percentage of untreated abscopal tumors inhibited by the indicated treatment of the primary tumor (4*p < 0.0001, 3*p < 0.001, 2*p < 0.01, 1*p < 0.05).

As shown in Fig. 7, the i.t. injection of aOX40 alone partially inhibited both treated and untreated tumor growth. However, when it was combined with NPS treatment, the growth of the untreated abscopal tumors was inhibited in 80% of the mice (Fig. 7A, B). NPS treatment alone only inhibits abscopal tumor growth in 10% of the treated mice, and aOX40 alone inhibits 50% of the abscopals. If the combination was additive, we would expect 60% inhibition, but we actually observed 80%, suggesting a synergistic effect when combined.

## Discussion

The most significant result from this study is the strong inhibition of abscopal tumor growth observed when the i.t. injection of aOX40 immediately followed NPS tumor treatment. This result suggests that the NPS-generated immune response may have translational benefits in the clinic by stimulating the immune system to seek out distant tumor sites and potentially metastases. This effect is known as the abscopal or “bystander” effect and it is a mechanism that could help clear tumor cells out of locally advanced sites without direct treatment.

We initially hypothesized that the combination of NPS and a TLR adjuvant, which was capable of preventing and/or inhibiting rechallenge tumor growth, would also inhibit the growth and/or cause regression of an abscopal tumor. However, that was not the case. The condition that showed significant synergy with NPS and was responsible for the greatest inhibition of abscopal tumor growth was the injection of aOX40 i.t. immediately following NPS treatment. We hypothesize that NPS triggers the initial immune recognition response through the release of Danger-Associated Molecular Patterns (DAMPs) and downstream signaling events, whereas the addition of aOX40 sustains a robust type 1 CD8+ T cell-mediated immune response by depleting the environment of regulatory T cells^36–38^ and promoting the survival and migration of cytotoxic T cells to distal tumor sites.^29,39–42^

Another interesting point is that the stronger NPS treatment of 360 mJ/mm^3^ combined with RES was not as effective as 180 mJ/mm^3^ in inhibiting the rechallenge tumor. This suggests that the lower-energy NPS treatment results in a stronger immune response than the higher-energy treatment.

### Limitations of this study

While the current findings are promising, it is important to keep in mind that these tumors were inoculated into the intradermal space, not into pancreatic tissue. Pan02 tumors exhibit a similar resistance to many chemotherapeutics as human PDAC tumors.^19,28^ However, they do not share the level of desmoplasticity or resistance to checkpoint blockade with human tumors,^43^ particularly when they are grown outside of the pancreas.^44,45^ Positive next steps would be to inoculate tumors orthotopically into the pancreas and allow these tumors to advance normally into surrounding tissues, although this procedure would be more variable as it is a more complex surgical procedure that may present unique hurdles. It is interesting to note, however, that aCD40 and aOX40 were shown to sensitize mice bearing orthotopically implanted Pan02 tumors to aPD-1/PD-L1,^43–45^ and thus, our NPS combinatorial strategy has supporting evidence.

Some other investigations that would have strengthened our article would be to monitor the maturation and migration of dendritic cells in the tumor and lymph nodes, as well as examining lymphocyte and myeloid cell populations in the blood plasma following NPS treatment and injection of immune modulators. Measuring the exhausted T lymphocyte population in the primary and secondary tumors would tell us whether immune checkpoint blockade therapy should be given.

Applying NPS to human PDAC in the clinic is feasible due to advances in endoscopic ultrasound (EUS). EUS with the addition of fine needle aspiration has been established over the past two decades as the premier diagnostic tool for staging many gastrointestinal luminal cancers, evaluating intramural lesions, and assessing the immediate extraluminal space.^46^ More recently, the role of EUS has been shifting from a diagnostic tool to an important therapeutic tool for treating mainly pancreatobiliary disorders. While EUS was initially used to inject ethanol^47^ or chemotherapeutic agents,^48^ in recent years its applications for treating pancreatic diseases have broadened, including the implementation of radiofrequency ablation and irreversible electroporation,^49^ traditionally used for treating solid tumors. EUS provides the highest image resolution among all the imaging modalities for pancreatic cancer because the ultrasound transducer is positioned against the inner wall of the duodenum on which the pancreas is tightly bound only millimeters away. Once the EUS detects the tumor location it is easy to utilize the working channel to deliver NPS to the tumor, as well as injecting adjuvants into the tumor with an injection needle. Unlike surgical approaches, EUS is minimally invasive and can be performed as an outpatient procedure with a low risk of complications. This approach may be the best for applying NPS therapy to the pancreatic tumors followed by the injection of immune stimulants into them.

## Conclusions

Pancreatic cancer presents both researchers and clinicians with significant therapeutic challenges due to its difficult TME and the probability of local advancement at initial diagnosis. NPS used in conjunction with immune enhancement with aOX40 may be quite effective in treating pancreatic tumors, as this combination not only induces cell death at the primary site but also may assist in the priming, expansion, and survival of antitumor CD8+ T cells, thus prompting their migration and infiltration into distal sites of locally advanced disease. In conclusion, NPS therapy may provide some of the key missing pieces required to provide a truly efficacious treatment of pancreatic tumors, expanding the repertoire of therapeutic options for this disease.
